# Evaluation of safety and efficacy of cyclosporine-A eye drops in herpetic stromal keratitis: a prospective randomized clinical trial

**DOI:** 10.1186/s12348-026-00573-2

**Published:** 2026-03-05

**Authors:** Nancy M. Lotfy, Hamad Alshatti, Rola Y. Khachaba, Sherein M. Hagras

**Affiliations:** 1 Ophthalmology Department, Faculty of Medicine, Cairo University, Cairo, Egypt; 2 Ophthalmology Department, Farwanyia Hospital, Sabah Al Nasser, Kuwait; 3 Department of Biological Sciences, University of Toronto, Toronto, ON Canada; 4 Ophthalmology Department, Faculty of Medicine, Mansoura University, Mansoura, Egypt

**Keywords:** Herpetic stromal keratitis, Cyclosporine, Prednisolone

## Abstract

**Purpose:**

To evaluate the additive effect of topical cyclosporine A (Cs-A) 0.05% eye drops with topical prednisolone compared with that of topical prednisolone acetate 1% eye drops alone for the treatment of herpetic stromal keratitis (HSK).

**Methods:**

Patients diagnosed with HSK were randomly divided into 2 groups: Group A received Cs-A eye drops b.i.d. with prednisolone eye drops q.i.d.; Group B received topical prednisolone q.i.d. with placebo eye drops b.i.d.; both Groups A & B received a systemic acyclovir therapeutic dose of 400 mg five times daily, tapered to twice daily on the 10th day. Follow-up was performed weekly for 2 months. The primary outcome measures included duration of treatment, corneal edema (CE), haziness (CH), punctate epitheliopathy (CP) and vascularization (CV). The secondary outcome measures included best corrected visual acuity (BCVA) and intraocular pressure (IOP).

**Results:**

Thirty eyes from 30 patients were included (15 in each group). At baseline, only the CP score was significantly greater in Group B. The duration of treatment was significantly shorter in Group A. At the 2-month follow-up, there were significant improvements in the CE, CH and CP scores in both groups compared with the baseline scores, but there were no significant differences between the groups. There was a significant improvement in BCVA in both groups compared with baseline scores, but there was no significant difference between the groups. Two patients in group B required topical drops to control the IOP.

**Conclusion:**

The addition of Cs-A eye drops to the treatment protocol for HSK patients has a significant role in shortening the duration of treatment in the management of HSK patients with and without epithelial ulceration.

**Registration:**

The study protocol was approved by the Local Review Board **(Registration code: 2261)**, and ClinicalTrials.gov **(ID: NCT05720715)**.

## Introduction

Herpetic stromal keratitis (HSK) is a combination of the local toxic effects of the virus with the host’s immunological response [1], accounting for 20–48% of all ocular herpes simplex virus (HSV) infections [2]. Patients who develop stromal disease are at increased risk of recurrence with a greater risk of permanent loss of vision due to deep corneal scarring and stromal vascularization. Although stromal inflammation is the hallmark of HSK, the exact pathogenesis is still unclear. HSK is a type of corneal immune-mediated inflammation, with CD4 + T lymphocytes playing a crucial role. In addition, the levels of interleukin-2 (IL-2) and gamma-interferon (IFN-γ) increase during the acute phase of infection. Taken together, these mediators are involved in stromal destruction [3, 4]. 

The standard paradigm for treating HSK involves the use of antivirals with topical immunosuppressants, mainly corticosteroids, aimed at dampening inflammation and hence preventing subsequent scar formation [5]. In this context, the Herpetic Eye Disease Study (HEDS) revealed that half of the treatment failures occurred within 6 weeks of steroid cessation; thus, very slow tapering over a prolonged period is recommended [6]. 

Although long-term topical corticosteroids are considered the customary treatment, they may be limited by ocular complications, such as possible recurrence of herpetic infection, high intraocular pressure (IOP), glaucoma development, and even resistance to treatment [7]. 

Cyclosporine-A (Cs-A) is a potential substitute for corticosteroids. Cs-A is an immunosuppressant that selectively targets T cells, blocking their proliferation as well as the production of interleukin-2 (IL-2) [8]. The clinical efficacy of Cs-A has been described for the treatment of numerous ocular surface inflammatory diseases, such as dry eye syndrome and vernal and atopic keratoconjunctivitis, with satisfying outcomes [9]. 

The present study was designed to evaluate the additive effect of topical 0.05% Cs-A eye drops with topical prednisolone compared with that of topical prednisolone acetate 1% eye drops alone for the treatment of HSK.

### Patients and methods

A prospective randomized controlled double-blinded clinical trial was conducted between February 2022 and November 2023 on 36 patients with HSK (36 eyes) at a tertiary ophthalmic hospital. The study protocol was approved by the local review board (Registration code: 2261) and ClinicalTrials.gov (ID: NCT05720715). Informed consent was obtained from each enrolled patient after the possible adverse effects of each therapeutic protocol were clarified. Ethical considerations were fulfilled in accordance with the tenets of Helsinki’s Declaration.

Adult patients with active HSK, diagnosed on the basis of their clinical presentation via slit lamp examinations, were enrolled in this study [10]. All patients had a history of previous attacks of herpetic epithelial keratitis with typical dendritic ulcers. The signs of HSK included corneal edema, infiltration, and vascularization with or without ulceration.

Only patients who did not use systemic acyclovir or topical steroids were selected. Patients with other types of keratitis were excluded from the study. Patients with a history of prior corneal surgery or ocular trauma, pregnant or lactating female patients and patients who received systemic or topical immunosuppressive drugs within one month of the study were also excluded.

The selected patients were randomly divided into 2 equal groups via the block randomization method. No patients presented with bilateral involvement. The first group (A) received Cs-A 0.05% eye drops *b.i.d.* together with prednisolone acetate 1% eye drops one drop *q.i.d*. Group B received topical prednisolone acetate 1% eye drops one drop *q.i.d.* with placebo eye drops (tear replacement) *b.i.d*. The 2 groups received oral acyclovir therapeutic dose 400 mg five times daily, which was then tapered to twice daily after 10 days of treatment. Patients with HSK with ulceration received topical prednisolone acetate (1% *b.i.d*.), and the dose was increased after healing of the ulcer to *q.i.d*. Topical prednisolone acetate eye drops 1% was modified according to patients’ response and clinical findings. The patients and examiners involved in the study were masked to the contents of the eye drops.

Before initiating the treatment and weekly for 2 months after treatment, the following parameters were recorded and compared between both groups. Slit-lamp examination, corneal haziness (CH), edema (CE), vascularization (CV) and punctate epitheliopathy (CP) were documented via digital corneal photographs. Intraocular pressure (IOP) was measured via a Goldmann applanation tonometer (Haag-Streit, Switzerland), and best-corrected visual acuity (BCVA) was measured via Snellen’s E chart (converted to LogMAR for statistical analysis). The primary outcome measures included duration of treatment, changes in corneal edema, and the development of corneal scarring or vascularization. The secondary outcome measures included changes in BCVA and IOP and any reported complications related to the treatment.

For statistical analysis, a scoring scheme was used to grade the corneal findings. For corneal edema, a score of 1 was used to describe mild corneal edema (minimal loss of transparency), a score of 2 was used for moderate edema (involving less than the anterior half of the corneal thickness), and a score of 3 was used for severe edema (involving more than half the corneal thickness). For corneal haziness, mild (iris details are still visible) = 1, moderate (iris details not clearly visible) = 2 and severe (anterior chamber structure not visible) = 3. Corneal punctate epitheliopathy was graded after fluorescence staining as follows: mild (scattered staining) was given a score of 1; moderate (confluent staining) = 2; and severe (compact staining) = 3. A corneal vascularization score of 0 indicates no evidence of corneal vascularization: 1 = one quadrant, 2 = two quadrants of corneal vascularization, and 3 = three or more quadrants of corneal vascularization.

### Statistical analysis

The data were analyzed via SPSS software v.16.00. Quantitative variables are presented as the means ± standard deviations (SDs), whereas categorical variables are presented as numbers and percentages. Kolmogorov–Smirnov tests were run to test the normal distribution of the data. For normally distributed variables, an independent t test was applied. The variables without a normal distribution were compared via the Mann‒Whitney and Kruskal‒Wallis tests. The chi-square test was applied to compare categorical variables. Intergroup analysis refers to comparing the data between the 2 groups. Intragroup analysis refers to comparing the data within the same group between the baseline and posttreatment values. The mean changes were used to describe the difference between the values at the end of the follow-up and the baseline values within each group.

For all the tests used, *p* ≤ 0.05 was considered statistically significant.

## Results

### Baseline data

The data were evaluated on 30 eyes of 30 patients allocated to 2 equal groups (15 each). Six patients did not complete the follow-up period and were not included in the results. The mean ± SD age of the patients was 41.7 ± 11.8 years (range: 22–80), with no significant difference between the two groups. Almost all the patients in Group B were males (93.3%), which was significantly greater than that in Group A (*p* = 0.007, chi-square test). Pretreatment corneal punctate epitheliopathy was significantly greater in group B (*p* < 0.001). The number of patients with ulceration in Group A was 10, and that in Group B was 9. The baseline demographic and clinical data are summarized in Table 1.

**Table 1:** Baseline characteristics among the studied groups

Pre-treatment Parameter	Group A *n* = 15 eyes	Group B *n* = 15 eyes	*P* value
Age(years) Mean ± SD/ range	38.4 ± 2.5/ (29–52)	45.1 ± 3.4/ (22–80)	0.187
Eye			
Right	3	8	0.06
Left	12	7	
Gender			
Male	7	14	0.007*
Female	8	1	
BCVA (log MAR) Mean ± SD	0.54 ± 0.32	0.55 ± 0.33	0.935
IOP mmHg Mean ± SD	16.3 ± 5.5	18.3 ± 8.6	0.539
Corneal edema (CE) Mean ± SD	1.86 ± 0.27	1.9 ± 0.78	0.87
Corneal haziness (CH) Mean ± SD	1.73 ± 0.56	1.6 ± 0.63	0.567
Corneal vascularization (CV) Mean ± SD	0.53 ± 0.99	0.4 ± 0.91	0.775
Corneal punctate epitheliopathy (CP) Mean ± SD	0.33 ± 0.89	2.06 ± 0.96	< 0.001*
With epithelial ulceration Without epithelial ulceration	10 5	9 6	1.0
Follow up period (weeks) Mean ± SD/range	11.2 ± 2.04/8–14	11.3 ± 2.2/8–14	0.932

Group A= treated by combination cyclosporine and prednisolone acetate eye drops. Group B= treated by prednisolone acetate eye drops alone; BCVA= best corrected visual acuity; Log MAR= logarithm of the minimum angle of resolution; IOP= intraocular pressure; Mann Whitney U test used for non-parametric continuous data. Chi square test used for categorical data. Significance at *p* ≤ 0.05*

### Primary outcome measures

#### Intergroup analysis

The mean duration of treatment was significantly shorter in Group A than in Group B (*p* = 0.03). The mean duration of treatment in group A was 10.29 ± 4.48 days, and that in group B was 17.5 ± 12.11 days. The mean CE scores were lower in Group A than in Group B, but the difference was not statistically significant (*p* = 0.389). The mean decrease in the CE score was greater in Group A than in Group A, but the difference was not significant (*p* = 0.557). The mean CH scores were lower in group A, but the difference did not reach statistical significance (*p* = 0.202). Compared with Group B, Group A revealed a statistically significantly greater mean decrease in CH (*p* = 0.035). No significant changes were detected in the means of the CV scores between the groups in either the posttreatment values (*p* = 0.256) or the mean change (*p* = 0.081). The mean CP scores continued to be significantly higher in Group B than in Group A at 2 months post-treatment (*p* = 0.002).

#### Intragroup analysis

Compared with the baseline values, corneal edema (CE) decreased significantly at 2 months within each group (*p* < 0.001). Both groups exhibited significant improvements in CH at 2 months compared with their baseline values (*p* = 0.001 in Group A and 0.034 in Group B). Compared with the baseline scores, the CV scores did not differ significantly between the two groups (*p* = 0.102 in group A and 0.564 in group B). However, there was a significant improvement in the mean CP score within Group B at the end of the follow-up period (*p = 0.012*).

### Secondary outcome measures

#### Intergroup analysis

The comparison of the mean ± SD logMAR BCVA values between the groups revealed no significant differences at the end of the follow-up period (*p* = 0.088). However, visual recovery was faster in group A. Additionally, the mean change in BCVA was not significant between the two groups (*p* = 0.376). The results of the IOP measurements revealed that the posttreatment values and the changes in IOP did not differ significantly between the 2 groups (*p* = 0.87 and 0.436, respectively). The recurrence rate was significantly greater in group A (*p* = 0.05). The treatment was stopped after the patient improved in terms of ulcer healing, corneal edema, infiltration and vascularization. Cs-A did not cause any significant complications. However, three patients experienced a temporary burning sensation after using the drops, but this did not lead to the discontinuation of the medication. Table 2 summarizes the posttreatment clinical findings and the changes within each group.

**Table 2:** Clinical findings in the 2 studied groups pre and post-treatment

Parameters Mean ± SD	Group A *n* = 15 eyes	Group B *n* = 15 eyes	P2 value
BCVA (log MAR)			
Pretreatment	0.54 ± 0.32	0.55 ± 0.33	0.935
Posttreatment	0.14 ± 0.14	0.23 ± 0.13	0.088
Mean change	0. 4 ± 0.25	0.32 ± 0.24	0.376
* P*1 value	0.001*	0.001*	-
IOP (mmHg)			
Pretreatment	16.3 ± 5.5	18.3 ± 8.6	0.539
Posttreatment	14.9 ± 3.86	14.27 ± 2.12	0.870
Mean change	1.3 ± 1.8	4.0 ± 8.8	0.436
* P*1 value	0.017*	0.197	-
Corneal edema (CE)			
Pretreatment	1.86 ± 0.27	1.9 ± 0.78	0.87
Posttreatment	0.13 ± 0.52	0.33 ± 0.62	0.389
Mean change	1.7 ± 0.46	1.6 ± 0.74	0.557
* P*1 value	0.001*	0.001*	-
Corneal haziness (CH)			
Pretreatment	1.73 ± 0.56	1.6 ± 0.63	0.567
Posttreatment	0.87 ± 0.74	1.2 ± 0.68	0.202
Mean change	0.87 ± 0.51	0.4 ± 0.63	0.032*
* P*1 value	0.001*	0.034*	**-**
Corneal vascularization (CV)			
Pretreatment	0.53 ± 0.99	0.4 ± 0.91	0.775
Posttreatment	0.2 ± 0.77	0.5 ± 0.83	0.250
Mean change	0.33 ± 0.72	-0.07 ± 0.46	0.083
* P*1 value	0.102	0.564	-
Corneal punctate epitheliopathy (CP)			
Pretreatment	0.33 ± 0.89	2.06 ± 0.96	< 0.001*
Posttreatment	0.13 ± 0.52	1.07 ± 0.88	0.002*
Mean change	0.2 ± 0.77	1.0 ± 1.13	0.032*
* P*1 value	0.317	0.012*	-
Duration for treatment (weeks)	1.47 ± 0.64	2.5 ± 1.73	0.03*
Number of recurrences	0.67 ± 0.72	0.13 ± 0.35	0.05*

Group A= treated by combination cyclosporine and prednisolone acetate eye drops. Group B= treated by prednisolone acetate eye drops alone; BCVA= best corrected visual acuity; Log MAR= logarithm of the minimum angle of resolution; IOP= intraocular pressure; Mann Whitney-U test used for non-parametric continuous data. Chi square test used for categorical data. Significance at *p* ≤ 0.05* Mean change: the change in each parameter compared to baseline values. P1 represents the intragroup comparison (comparing the post-treatment results to the baseline data) while P2 represents the intergroup comparison (comparing post-treatment results and mean change between the two groups)

#### Intragroup analysis

Within each group, the BCVA improved significantly compared with the baseline values (*p* = 0.001 in both groups). The IOP values significantly decreased in group A at the end of the follow-up period (*p* = 0.017). The decrease was not significant in group B (*p* = 0.197), in which 2 patients required topical antiglaucoma medications to control the IOP (combination of beta blockers and dorzolamide).

#### Subgroup analysis (HSK with epithelial ulceration)

Compared with eyes without ulceration, eyes that presented with stromal keratitis with ulceration were not significantly different at the end of the follow-up period in terms of BCVA, duration of healing, rate of recurrence or mean IOP (*p* = 0.24, 0.16, 0.22 and 0.82, respectively). In Group B, the posttreatment BCVA was significantly lower in patients with ulceration than in those without ulceration (*p* = 0.02). Additionally, the mean IOP was significantly greater in patients with ulceration (*p* = 0.013).

## Discussion

Among the thirty patients included in this study, twenty-one (70%) were males. Different studies in the literature have presented varying results on the incidence of herpetic keratitis based on sex. For India, for example, most studies reported a higher incidence in males [11]. Conversely, other studies reported a higher incidence in females [12]. A study conducted in France reported equal incidences of both sexes [13]. 

The mean ± SD of the patients’ age in this study was 41.7 ± 11.8 years, which is comparable to the mean age of patients reported in other studies [11, 14, 15]. 

Our study revealed that the addition of topical Cs-A to the treatment protocol for HSK patients significantly shortened the duration of treatment. (The mean duration of treatment in group A = 10.29 ± 4.48 days, and that in group B = 17.5 ± 12.11 days). The average duration of treatment in group B is comparable to the estimated average duration of treatment for HSK disease in the literature, which is 17.6 days [16]. The addition of Cs-A to the treatment protocol in group A significantly shortened the duration of treatment, not only compared with that in group B but also compared with the average estimated duration of treatment reported in the literature.

Moreover, this study reports that posttreatment clinical outcomes of HSK, although statistically comparable in both groups, have better results in patients treated with Cs-A. Furthermore, the intragroup analysis revealed that the posttreatment clinical outcomes (including BCVA, duration of treatment, IOP and rate of recurrence) are statistically comparable in patients with keratitis with and without ulceration in group (A) However, these outcomes are significantly lower in patients with keratitis with ulceration than in those with keratitis without ulceration in group (B) This result suggests that adding Cs-A to the treatment protocol of HSK patients with ulceration, although few patients were involved in our study, helps improve the clinical outcomes to be comparable with those of HSK patients without ulceration.

A randomized clinical trial (on 38 eyes with HSK) conducted by Peyman A et al. in 2019 suggested that both 2% Cs-A and 1% prednisolone acetate eye drops are effective for the treatment of HSK [17]. Rao (2006), Heiligen-haus and Steuhl (1999), and Gunduz and Ozdemir (1997) reported the effectiveness of Cs-A in the management of non-necrotizing HSK [5, 18, 19], with improvement in stromal inflammation in 83% of cases [20]. However, the aforementioned studies did not explore the effect of Cs-A in patients with HSK with ulceration.

Topical cyclosporin has shown a promising role in the management of recurrent corneal erosion syndrome and refractory epithelial defects in various ocular diseases [21]. 

Cs-A suppresses the activity of T cells, interferes with the induction of cytokines and other inducible genes that are required for the immune response, and inhibits angiogenesis. As a result, it decreases access to the corneal stroma for T cells [22]. Topical steroids represent the keystone in the treatment of HSK. However, prolonged use of steroid eye drops is associated with a greater risk of ocular complications.

To the best of our knowledge, this is the first study to discuss the adjunctive effect of topical Cs-A and its role in shortening the duration of treatment and improving clinical outcomes in both HSK patients with and without ulceration.

Two patients in group B recovered from antiglaucoma to control IOP, and the increase in IOP in this group could be due to the longer duration of topical steroid use; another possible etiology is the persistence of inflammation that is unresponsive to steroid treatment [18]. 

Our results showed that the CV scores did not differ significantly between the two groups and the baseline values. The short follow-up duration could have contributed to this result. Moreover, the clinical grading system of the CV in our study depended only on the number of involved quadrants of the cornea. A more detailed clinical grading system could help better identify changes in corneal vascularization.

In our study, Cs-A did not cause any significant complications, apart from a temporary burning sensation after using the drops in three patients. However, this did not lead to the discontinuation of the medication. A study conducted by Heiligenhaus and Steuhl (1999) reported toxic epitheliopathy from topical Cs-A in six out of 18 patients with HSK [5]. 

A higher recurrence rate in group A was reported in the present study. Nevertheless, all patients who presented with recurrences in both groups had a history of noncompliance with systemic antiviral prophylaxis. Most of the morbidity associated with ocular HSV is due to HSK. Several clinical trials have investigated the role of systemic antiviral prophylaxis in preventing HSK recurrence [23, 24]. Long-term low-dose oral antiviral prophylaxis is recommended for patients with a history of recurrent HSK [25]. 

The small sample size is the main limitation of this study, especially when comparing the clinical outcomes of HSK patients with and without ulceration within each group. Additionally, patients were lost to follow-up as soon as they experienced improvement in their ocular symptoms. Those patients were excluded from the study, which added to the small sample size. Large-scale clinical trials are needed to better determine the role of Cs-A eye drops as an adjunct or alternative to topical steroids in the treatment of HSK.

The clinical grading system for the evaluation and follow-up of corneal vascularization is deficient. A more comprehensive grading score could help better evaluate the vascularization state of the cornea. This score could include the number of involved clock hours instead of quadrants, the state of vessels if they are ghost vessels or active vessels, and the degree of invasion of the central cornea.

The incorporation of Cs-A eye drops into the treatment protocol for HSK patients has significantly shortened the duration of treatment for HSK patients with and without epithelial ulceration. It may also have a role in improving clinical outcomes. These effects could lessen both the human and economic burdens of this potentially blinding disease.

## Data Availability

All data supporting the findings of this study are available within the paper and its Supplementary Information.
